# miR-34a Inhibits Cell Proliferation by Targeting SATB2 in Hepatocellular Carcinoma

**DOI:** 10.1155/2018/2863902

**Published:** 2018-12-06

**Authors:** Gang Wu, Zhixi Li, Youyu Wang, Xueming Ju, Rui Huang

**Affiliations:** ^1^Department of Hepatobiliary Surgery, Sichuan Provincial People's Hospital, University of Electronic Science and Technology of China, Chengdu, Sichuan 610072, China; ^2^Department of Pediatric Surgery, Sichuan Provincial People's Hospital, University of Electronic Science and Technology of China, Chengdu, Sichuan 610072, China; ^3^Department of Thoracic Surgery, Sichuan Provincial People's Hospital, University of Electronic Science and Technology of China, Chengdu, Sichuan 610072, China; ^4^Department of Ultrasound, Sichuan Provincial People's Hospital, University of Electronic Science and Technology of China, Chengdu, Sichuan 610072, China

## Abstract

Hepatocellular carcinoma (HCC) is the most common type of malignancy of the liver and has been reported as the third most frequent cause of cancer associated death worldwide. Accumulating evidence showed that the expression of miR-34a was abnormal in HCC patients; however, the role of miR-34a in HCC is not clear. In this study, we have observed low expression of the miR-34a in both HCC tissues and hepatoma cell line as compared to normal control. Further to investigate the role of miR-34a in HCC development, HepG2 cells were transfected with miR-34a mimic. Following transfection, miR-34a expression was significantly increased, which further repressed proliferation of HepG2 cells. Bioinformatics, Luciferase Reporter, RT-qPCR, and western blotting assays indicated that special AT-rich sequence-binding protein-2 (SATB2) is a direct target of miR-34a in HCC cells. There was a negative correlation between the expression levels of SATB2 and miR-34a. Investigation into the molecular mechanism indicated that miR-34a regulated cell proliferation through inhibiting SATB2. Therefore, the results of the present study may improve understanding regarding the role of miR-34a in regulating cell proliferation and contribute to the development of novel therapy of HCC.

## 1. Introduction

Hepatocellular carcinoma (HCC) is believed to be one of the most common types of malignancy of the liver and has been reported as the fifth most prevalent cancer and third most frequent cause of cancer associated death worldwide [1, 2]. In spite of the therapeutic advances in diagnosis and clinical treatment, the 5-year survival rate of HCC across all cancer stages is still less than 30 % due to postoperative recurrence [3]. Therefore, it is urgent to study the underlying molecular mechanisms involved in the HCC progression and provide an effective therapeutic strategy for the treatment of HCC.

miRNAs are short noncoding RNAs molecules involved in gene regulation at a posttranscriptional level by binding to the 3'-untranslated regions of target mRNA to cause translational repression or mRNA cleavage and impact fundamental cellular processes, such as differentiation, metabolism, and metabolic stress [4, 5]. Increasing researches have indicated that many miRNAs, including miR-34a [6], miR-211 [7], and miR-873 [8], are aberrantly expressed in HCC samples and exerted an effect in cell proliferation, apoptosis, and metastasis through regulating target mRNAs gene. Previous studies have shown that the miR-34a could affect the proliferation and apoptosis of hepatocellular carcinoma by regulating HDAC1 [9], c-Met [6], and SIRT1 [10]. At present, only Jiang G et al. reported the expression level of SATB2 in hepatocellular carcinoma [7]. Xin G E et al. reported that the miR-34a inhibited the proliferation, migration, and invasion of oral squamous cell carcinoma by downregulating SATB2 expression [11]. However, whether miR-34a can inhibit the proliferation of hepatocellular carcinoma by regulating SATB2 has not been reported.

In this study, we aimed to identify the role of miR-34a in cell proliferation in human hepatoma cell line HepG2 and investigate the molecular mechanism of miR-34a-regulated cell proliferation. In the end, we found that miR-34a was lowly expressed in HCC tissues and HCC cell line. And the overexpression of miR-34a could inhibit cell proliferation in HepG2 cells through inhibiting SATB2.

## 2. Materials and Methods

### 2.1. Tissue Samples

The total samples of 12 cases of hepatocellular carcinoma tissues and 8 cases of hepatocellular carcinoma adjacent tissues were from patients who underwent surgery in Sichuan Provincial People's Hospital from 2016 to 2017. The present study was approved by the Ethics Review Board at the University of Electronic Science and Technology of China, and written informed consent was obtained from all patients.

### 2.2. Cell Culture

The human hepatoma cell line HepG2 and hepatocyte cell line HL7702 were obtained from purchased from Procell Life Science & Technology Co. Ltd (Wuhan, China). The cells were incubated and cultured in Dulbecco's modified Eagle's medium (DMEM; Hyclone, UT, USA) supplemented with 10% Fetal Bovine Serum (FBS; Gibco, CA, USA). Cells were maintained at 37°C in an incubator with 5% CO_2_. For subculturing purpose, cells were incubated with 0.25% trypsin at 37°C, and cultures were used at 80% confluency.

### 2.3. Transfection

The miR-34a mimic and negative control molecules (NC mimic) were purchased from Guangzhou RiboBio Co., Ltd. The siRNA against SATB2 (SATB2-siRNA) and negative control siRNA (NC-siRNA) were chemically synthesized by Shanghai GenePharma Technology Co., Ltd. Cell transfection was performed using Lipofectamine® 2000 (Invitrogen, CA, USA) according to the manufacturer's protocol with 50 pmol/ml miR-34a mimic and negative control molecules, or 40 pmol/ml SATB2-siRNA and NC-siRNA. 24 h before transfection, cells were seeded into a 6-well plate at a density of 1x10^5^ cells/well and then transfected until the cells were in the log phase. Fresh medium was replaced after 6 h. After transfection of 24-48 h, cells were collected for RT-qPCR or western blotting analyses. The SATB2-siRNA and NC-siRNA sequences were shown as follows: SATB2-siRNA sense strand 5′-GUC AGA GAU GAG CUG AAG ATT-3′ and antisense strand 5′-UCU UCA GCU CAU CUC UGA CTT-3′; NC-siRNA sense strand 5′-UUC UCC GAA CGU GUC ACG UTT-3′ and antisense strand 5′-ACG UGA CAC GUU CGG AGA ATT-3′.

### 2.4. Reverse Transcription-Quantitative Polymerase Chain Reaction (RT-qPCR)

Relative miR-34a and SATB2 mRNA expression were routinely detected by RT-qPCR. Briefly, total RNA was extracted from tissues or cell lines using Trizol reagent (Invitrogen, CA, USA) according to the manufacturer's instruction. Then, the PrimeScript™ RT Reagent Kit (TaKaRa, Dalian, China) was applied to transcribe the extracted total RNA. RT-qPCR was performed using SYBR Premix Ex Taq II (TaKaRa, Dalian, China) with the Applied Biosystems 7500 Real-Time PCR System (Applied Biosystems, CA, USA). The relative expressions of SATB2 were calculated using the 2^−ΔΔCT^ method and normalized to the housekeeping gene *β*-actin. The specific primer sequences were as follows: SATB2 forward, 5′-CCT GGC CCT GGG GTA TTC T-3′, and reverse, 5′-GTGCATCTGTCACATAACTGAGG-3′; *β*-actin forward, 5′-GAA GAT CAA GAT CAT TGC TCC T-3′, and reverse, 5′-TAC TCC TGC TTG CTG ATC CA-3′. For the quantification of miRNA expression, the reverse transcription (RT) reaction was carried out using Bulge-Loop™ miRNA qRT-PCR Primer (RiboBio Co., Ltd, Guangzhou, China). RT reaction was processed at 42°C for 60 min and 70°C for 10 min. Gene expression levels were quantified at 95°C for 10 min, followed by 40 cycles of 95°C for 2 s, 60°C for 20 s, and 70°C for 10 s. U6 served as the internal control.

### 2.5. Cell Viability by CCK-8 Assay

Cell proliferation was evaluated using a Cell Counting Kit-8 (CCK-8; Dojindo Molecular Technologies, Inc., Kumamoto, Japan). HepG2 cells (5×10^3^/ well) were suspended and cultured in 96-well plates overnight. Subsequently, cells were transfected with miR-34a mimic or SATB2-siRNA sequence and incubated for 12, 24, and 36 h. 10% CCK-8 (Biosharp, Anhui, China) diluted in fresh medium was added to each well with a further incubation for 1 h. The absorbance at 450 nm was measured using a microplate reader (Thermo Fisher Scientific, Inc.) to generate cell growth curves.

### 2.6. Luciferase Reporter Assay

MiR-34a targets were predicted using bioinformatics software including TargetScan (http://www.targetscan.org/), mirDB (http://mirdb.org/), and DIANA TOOLS (http://diana.imis.athena-innovation.gr). HepG2 cells (3x10^4^/well) were seeded into 24-well plates followed by transfection with the Renilla Luciferase pRL-TK plasmid plus the Recombinant Firefly Luciferase pGL3 Reporters containing 3' UTR region of human SATB2 (GenePharma Technology Co., Ltd, Shanghai, China) in combination with miR-34a mimic and NC mimic by using Lipofectamine® 2000. 24 h after transfection, cells were collected and lysed. Luciferase and Renilla signals were measured using a Dual-Luciferase Reporter Assay Kit (Promega, WI, USA) according to the manufacturer's instructions.

### 2.7. Western Blotting Assay

Cells specimens were lysed using RIPA lysis buffer (Boster, Wuhan, China). Subsequently, protein samples (20 *μ*g) were separated by 10% SDS-PAGE gel and then transferred onto a PVDF membrane (Millipore, MA, USA) via a Bio-Rad II System (Bio-Rad Laboratories, Inc.). Then the membranes were blocked with 5% skim milk powder at room temperature for 1 h and incubated with a rabbit monoclonal antibody against STAB2 and *β*-actin (1:500; Cell Signaling Technology, MA, USA) and goat anti-rabbit IgG. Protein bands were detected by an ECL chemiluminescence kit (Millipore, MA, USA) according to the manufacturer's instructions. Protein levels were calculated relative to *β*-actin.

### 2.8. Statistical Analysis

Statistical evaluation was performed using SPSS 20.0 software (SPSS, Inc., Chicago, IL, USA). Values were presented as the means ± standard deviation (SD) from three separate experiments. Student's t-test was used to determine the differences between two groups. One-way analysis of variance (ANOVA) was used to estimate the statistical differences between three groups. p < 0.05 was considered to indicate a statistically significant difference.

## 3. Results

### 3.1. Expression of miR-34a Is Decreased in HCC Tissues and Cell Lines

**Figure 1:**
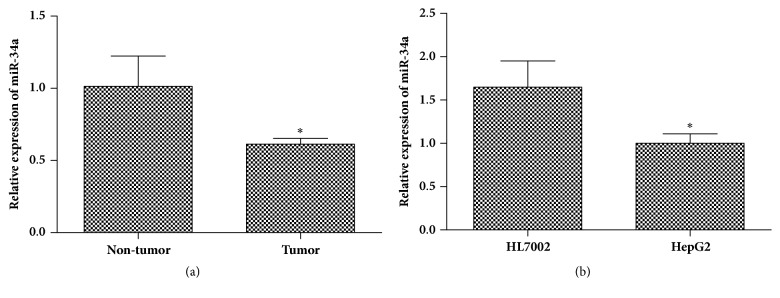
**Decrease of miR-34a expression level in HCC. **RT-qPCR was applied to quantify the expression level of miR-34a in HCC tissues and cell lines. (a) miR-34a expression in HCC tissues (n=12) and adjacent tissues (n=8), ^*∗*^*p* < 0.05 versus adjacent tissues. (b) Expression level of miR-34a in HepG2 and HL7702 cell lines, ^*∗*^*p* < 0.05 versus HL7702 cells.

To determine whether miR-34a was dysregulated in HCC, qRT-PCR was performed to evaluate the expression level of miR-34a in HCC tissues and HCC-adjacent tissues, hepatoma cell, and hepatocyte cell. As shown in Figure 1(a), the miR-34a mRNA was significantly downregulated in HCC tissues compared with adjacent tissues. Furthermore, we detected the expression level of miR-34a in HCC cell line (HepG2). The results showed that the miR-34a mRNA was markedly decreased in HepG2 cells compared with a normal liver cell line of HL7702 (Figure 1(b)). These data suggested that miR-34a expression was reduced in HCC.

### 3.2. Overexpression of miR-34a Inhibits Cell Proliferation

**Figure 2:**
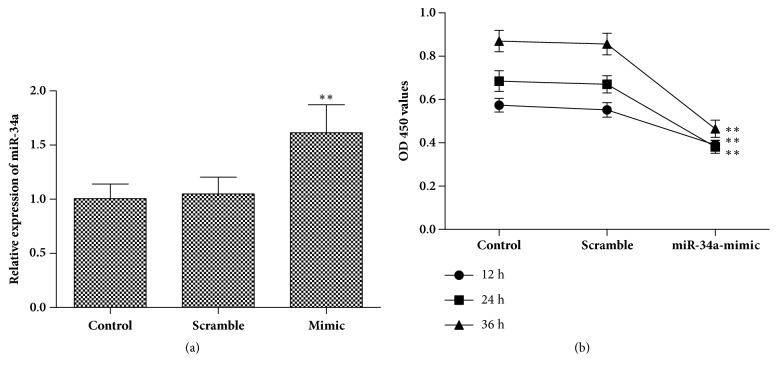
**The effects of miR-34a overexpression on cell proliferation. **(a) RT-qPCR was applied to quantify the expression level of miR-34a after transfection with miR-34a mimic or scramble control mimic (50 pmol/ml). (b) HepG2 cell proliferation rate was determined by a CCK-8 assay following transfection with miR-34a mimic or scramble control mimic (50 pmol/ml) for 12, 24, and 36 h. ^*∗∗*^*p* < 0.01 versus control group.

To investigate the biological function of miR-34a in HCC development, HepG2 cells were transfected with miR-34a mimic. RT-qPCR results showed that the miR-34a expression was significantly increased in miR-34a mimic group compared with control group, which indicated high transfection efficiency in HepG2 cells (Figure 2(a)). Furthermore, we detected cell proliferation after transfection. As shown in Figure 2(b), the upregulated expression of miR-34a inhibited the proliferation of HepG2 cells compared with control cells. These data demonstrate that the overexpression of miR-34a can effectively reduce the cell proliferation in HepG2 cells.

### 3.3. SATB2 Is a Direct Target of miR-34a in HCC Cells

**Figure 3:**
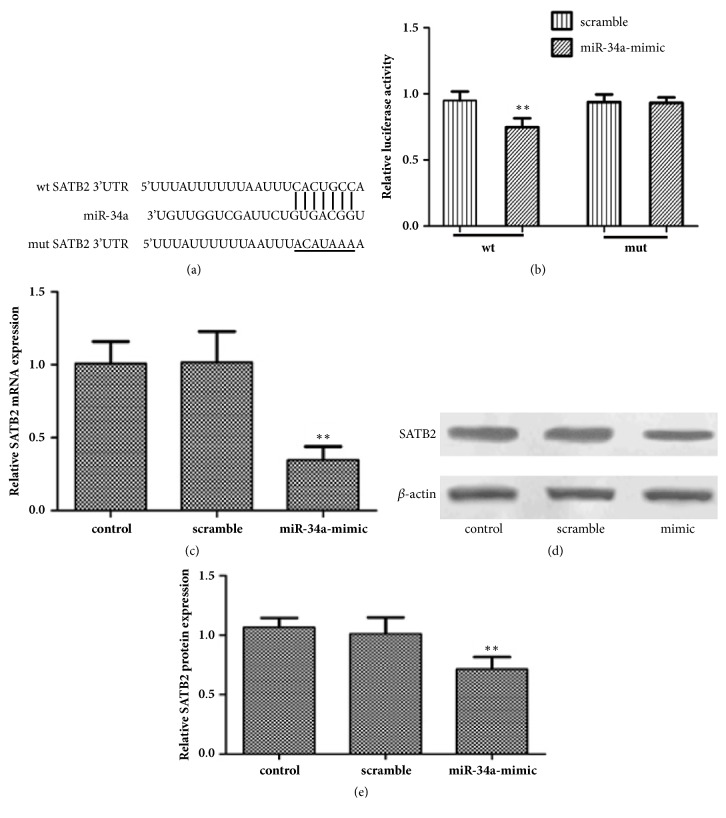
**miR-34a targets SATB2 in HCC cells. **(a) Predicted miR-34a target sites in the 3'UTR of STAB2. (b) The relative luciferase activities of STAB2-wt, STAB2-mut were measured using a Dual-Luciferase Reporter Assay Kit following transfection with miR-34a mimic (50 pmol/ ml). (c) RT-qPCR was applied to check the expression of SATB2 mRNA. (d) Western blotting was applied to check the expression of SATB2 protein. (e) The relative intensity of SATB2 protein was shown as a bar graph. ^*∗*^*p* < 0.05 and ^*∗∗*^*p* < 0.01 versus control group.

Using bioinformatics analysis, we found that SATB2 3'-UTR contained a conserved putative target site for miR-34a (Figure 3(a)). To validate the relationship between miR-34a and SATB2, we tested the luciferase activity, the mRNA, and protein level of SATB2 in HepG2 cells following transfection with miR-34a mimic. As shown in Figure 3(b), miR-34a mimic repressed the activity of Luciferase Reporters containing SATB2 3'-UTR. Meanwhile, the overexpression of miR-34a inhibited the mRNA expression of SATB2 (Figure 3(c)). Western blotting results also showed that transfection with miR-34a mimic significantly decreased the protein expression of SATB2 (Figures 3(d) and 3(e)). Taken together, we demonstrated that miR-34a targets SATB2 and suppresses its expression in HCC cells.

### 3.4. SATB2 Silencing Enhanced the Effect of miR-34a Mimic on Cell Proliferation Inhibition

**Figure 4:**
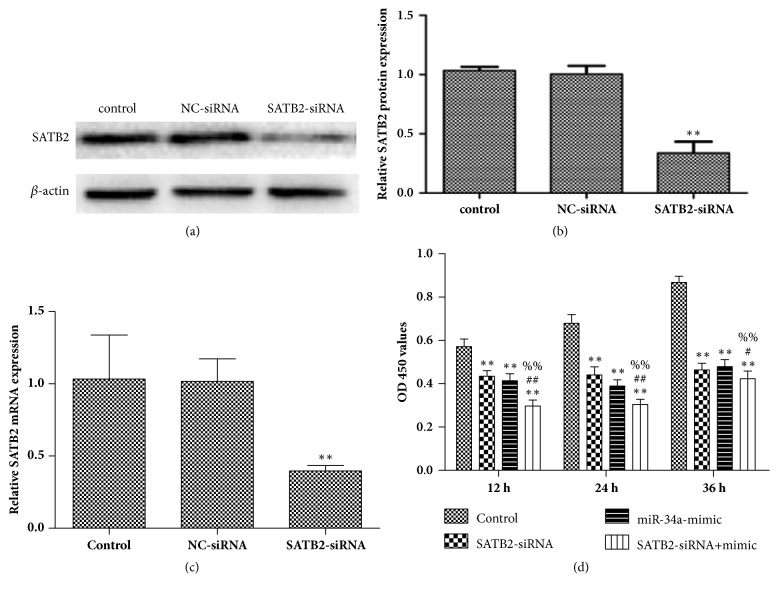
**miR-34a regulated cell proliferation through inhibiting SATB2. **(a) Western blotting was used to detect the protein expression of SATB2 in HepG2 cells following transfection with SATB2-siRNA sequence (40 pmol/ ml) for 36 h. (b) The relative intensity of SATB2 protein was shown as a bar graph. ^*∗*^*p* < 0.05, versus control group. (c) RT-qPCR was applied to check the expression of SATB2 mRNA following transfection with SATB2-siRNA sequence (40 pmol/ ml) for 24 h. ^*∗∗*^*p* < 0.01 versus control group. (d) HepG2 cell proliferation rate was determined by a CCK-8 assay following transfection with miR-34a mimic (50 pmol/ ml) and SATB2-siRNA sequence (40 pmol/ ml) for 12, 24, and 36 h. ^*∗∗*^*p* < 0.01 versus control group; ^#^*p* < 0.05 and ^##^*p* < 0.01 versus SATB2-siRNA group; ^%%^*p* < 0.01 versus miR-34a mimic group.

To determine whether SATB2 is involved in miR-34a regulated cell proliferation, we used a SATB2-siRNA sequence silencing SATB2. Western blotting results showed that the expression of SATB2 was significantly decreased following transfection with SATB2-siRNA sequence in HepG2 cells (Figures 4(a) and 4(b)). Meanwhile, the SATB2-siRNA sequence also inhibited the mRNA expression of SATB2 (Figure 4(c)). These data suggested the expression of SATB2 was successfully inhibited by SATB2-siRNA sequence. Further the CCK-8 results showed that the silencing of SATB2 inhibited the HepG2 cells proliferation. In addition, the silencing of SATB2 could enhance the effect of miR-34a mimic on cell proliferation (Figure 4(d)). These results indicated that miR-34a regulated cell proliferation through inhibiting SATB2.

## 4. Discussion

Increasing studies have highlighted the biological roles of deregulated miRNAs in almost all types of human cancer. There is a dual function of miRNAs, that is, tumor progression and negative regulation expression of their target mRNAs to play the role of oncogenes or tumor suppressor genes [12]. MiR-34 family is a kind of highly conserved miRNA, including three homologous genes, namely, miR-34a, miR-34b, and miR-34c. Among them, miR-34a is the result of miR-34 gene mutation, located on human chromosome 1p36 [13]. Studies have suggested that miR-34a is a tumor suppressor and is frequently lost or downregulated in most tumor tissues including HCC [14]. In addition, the miR-34a expression levels in serum could predict bone metastasis in hepatocellular carcinoma patients [15]. Thus, abnormal expression of miR-34a can be used as a marker of tumor risk, diagnosis, and prognosis in HCC patients [16]. Our study showed that miR-34a expression was down-regulated in HCC cells and HCC tissues, which is consistent with other studies. The overexpression of miR-34a can lead to proliferation and invasion inhibition in breast cancer [17], prostate cancer [18], osteosarcoma [19], and so on. To clarify the role of miR-34a in HCC, HepG2 cells were transfected with miR-34a mimic with high transfection efficiency. As expected, the overexpression of miR-34a markedly inhibited the proliferation of HepG2 cells. These data indicated that miR-34a may act as a tumor suppressor whose downregulation contributed to the progression and metastasis of HCC.

It is well known that the regulation of miRNA on cell biological behavior is based on its regulation of downstream target genes. SATB2 is a nuclear matrix-associated transcription factor and epigenetic regulator that is involved in transcription regulation and chromatin remodeling [20, 21]. Increasing researches have indicated that SATB2 was aberrantly expressed in a variety of malignant tumors [22, 23]. Jiang G et al. reported that SATB2 was highly expressed in HCC, and the overexpression of SATB2 promoted cell proliferation and metastasis [7]. Xin G E et al. reported that miR-34a inhibited the proliferation, migration, and invasion of oral squamous cell carcinoma by downregulating SATB2 [11]. However, the relationship between miR-34a and SATB2 in HCC is not clear. Using bioinformatics analysis, we found that the 3'-UTR of SATB2 contained a conserved putative target site for miR-34a in HepG2 cells. The Luciferase Reporter assay further confirmed the bioinformatics prediction. In addition, we found that the mRNA and protein levels of SATB2 in HepG2 cells were both decreased after transfection with miR-34a mimic, indicating that SATB2 is the downstream target gene of miR-34a, and the miR-34a could regulate the target gene not only at the posttranscriptional level, but also at the transcriptional level. Further to determine whether SATB2 is involved in miR-34a regulated cell proliferation, the HepG2 cells were transfected with SATB2-siRNA sequence. In our research, the low expression of SATB2 can not only inhibit cell proliferation, but also enhance the effect of miR-34a mimic on cell proliferation in HepG2 cells, indicating that miR-34a regulated cell proliferation through inhibiting SATB2 in HCC.

In summary, the current study indicates that the miR-34a exerts a tumor suppressive function in HCC by inhibiting cell proliferation. Additionally, we first found that miR-34a inhibited HCC progression by inhibiting SATB2. These findings are encouraging and suggest that miR-34a might be employed as a novel and effective therapeutic target for HCC in the future. However, the mechanism of miR-34a regulated HCC progression remains to be further researched.

## Data Availability

The datasets used or analyzed during the current study are available from the corresponding author on reasonable request.
